# Determining Reference Intervals and Median Blood Creatinine Levels in Children from Three Different Regional Populations

**DOI:** 10.3390/jcm14155373

**Published:** 2025-07-30

**Authors:** Ferdy Royland Marpaung, Hari Basuki Notobroto, Risky Vitria Prasetyo, Djoko Santoso, Etienne Cavalier, Aryati Aryati

**Affiliations:** 1 Doctoral Program of Medical Science, Faculty of Medicine, Universitas Airlangga, Surabaya 60132, Indonesia; ferdy.royland.marpaung-2022@fk.unair.ac.id; 2 Faculty of Public Health, Universitas Airlangga, Surabaya 60115, Indonesia; haribasuki.n@fkm.unair.ac.id; 3 Department of Child Health, Faculty of Medicine, Universitas Airlangga, Surabaya 60115, Indonesia; risky-v-p@fk.unair.ac.id; 4 Department of Internal Medicine, Faculty of Medicine, Universitas Airlangga, Surabaya 60132, Indonesia; djoko-santoso@fk.unair.ac.id; 5 Department of Clinical Chemistry, University of Liège, Centre for Interdisciplinary Research on Medicine, CHU Sart Tilman, 4000 Liège, Belgium; etienne.cavalier@chuliege.be; 6 Department of Clinical Pathology, Faculty of Medicine, Universitas Airlangga, Surabaya 60286, Indonesia

**Keywords:** creatinine, reference interval, Q creatinine, kidney function, non-communicable disease

## Abstract

**Background**: A critical gap exists in the current literature regarding pediatric-specific creatinine reference data. This study established age- and sex-stratified reference intervals and a corresponding median (Qcr) model for serum creatinine in children, providing a crucial foundation for improved diagnostic accuracy and clinical decision-making in this vulnerable population. **Methods**: A total of 9090 children (52.38% males and 47.65% females) who were getting regular check-ups at clinical laboratories in three regions were included in this study to establish Qcr serum and reference ranges for creatinine concentration. **Results**: The reference values and serum Qcr creatinine were established for children based on age and sex. Both males and females experience an incremental increase in creatinine levels with advancing age. In addition, significant differences were seen across the three areas in other age groups (*p* < 0.05). **Conclusions**: These newly established, age- and sex-stratified reference and Qcr values provide a critical resource for clinical laboratories, empowering clinicians to more accurately assess pediatric renal function and enabling more precise, individualized care for children with renal concerns.

## 1. Background

Chronic kidney disease (CKD) in pediatric populations represents a significant and growing health concern, with reported prevalence rates ranging from 12.1 to 74.7 cases per million children [1]. The complex pathophysiology of CKD in children manifests in a variety of clinical abnormalities, including compromised growth and nutritional status, electrolyte derangements, renal osteodystrophy, anaemia, and hypertension [2]. A critical factor contributing to the escalating CKD burden is the frequent sequelae of acute kidney injury (AKI), wherein extensive and irreversible nephron damage often precipitates the development and progression of CKD [3]. Consequently, the timely identification and intervention in cases of kidney injury are paramount for mitigating the advancement of CKD and ultimately averting progression to end-stage renal disease (ESRD) [4]. Precise assessment of pediatric renal function is paramount for the early detection and management of kidney injury. For instance, AKI could be established using baseline creatinine levels or a decreasing estimated glomerular filtration rate (eGFR) [5]. Currently, race-free eGFR is widely used; therefore, determining the median creatinine value (Qcr) is advantageous since it can be applied as a determinant in the creatinine-based race-free estimated glomerular filtration rate (eGFR) formula [6,7].

Serum creatinine, a well-established biomarker of renal health, serves as a crucial trigger for further diagnostic evaluation and therapeutic intervention, guiding clinicians in mitigating potential nephrotoxicity from medical interventions. These interventions may include careful monitoring of medication administration and optimization of hydration status [8]. Beyond its utility in detecting kidney injury, serum creatinine concentration plays a vital role in drug dosing and chronic kidney disease (CKD) management, enabling the estimation of glomerular filtration rate (eGFR) and facilitating personalized patient care [9,10,11].

The reference interval is crucial for physicians when interpreting laboratory test results, as it significantly influences assessments, including those related to creatinine examinations. The creatinine reference may differ in each population since creatinine levels are influenced by muscle mass, sex, age, race, etc. [12]. Hence, the objective of this study is to establish reference intervals for blood creatinine levels and the Qcr by utilizing data from children under 18 years of age.

## 2. Methods

### 2.1. Study Population

The individuals who took part in routine medical family screening at the Pramita Laboratories in Indonesia were chosen from 2019 to 2024 based on the following criteria: they had to be under 18 years old and have had a creatinine test. Exclusion criteria included individuals referred from the Nephrology Clinic or those sent by their nephrologist, those with a history of diabetes or who were sent from the diabetes clinic, and those who had a history of extremity amputation, kidney disease, and kidney failure. Data on creatinine concentration were retrospectively derived from laboratory information systems (LIS). Therefore, the lack of specific data to exclude conditions like professional bodybuilding or eating disorders, which significantly impact muscle mass and thus creatinine, might remain due to data availability.

Individual data originated from three distinct regions: Sumatera, Java, and Sulawesi. These represent three regional zones in Indonesia: Sumatera for the western area, Java for the middle region, and Sulawesi for the eastern region.

Reference intervals were established using an indirect method [5], which involved the retrospective analysis of serum creatinine data obtained from the LIS of individuals undergoing routine medical family screening at Pramita Laboratories in Indonesia.

The study was conducted in accordance with the Declaration of Helsinki (as revised in 2013). The Dr. Soetomo Academic General Hospital Institutional Review Board (No. 0934/KEPK/III/2024) accepted this research project. The Ethics Committee waived the need for consent because our study was considered retrospective. The authors have no access to individual identity during and after collecting the data.

### 2.2. Serum Creatinine Examination

Creatinine serum concentration was measured using enzymatic techniques via an automated chemistry analyser (Architect Abbott, Irving, TX, USA). Every single test was tracked using an IDMS calibrator (calibrator number 08P65/6K30, Abbott, Irving, TX, USA), with weekly calibration conducted. Two levels of Lyphochek-assayed chemical quality control materials (Bio-Rad, Hercules, CA, USA) were tested twice daily to ensure internal quality control in creatinine measurement. Throughout the study period, the serum creatinine test had a coefficient of variation (CV), demonstrating within-laboratory precision, of 1.19% and 0.67% at QC levels 1 and 2, respectively. From 2019 to 2024, the laboratory took part in the Indonesian Association of Quality Assurance for Clinical Laboratory’s external proficiency testing programme (BBLK), and the findings were all good (Z-scores between 0.14 and 1.09%).

### 2.3. Statistical Analysis

Blood creatinine concentration data were assessed for normality using the Kolmogorov–Smirnov test. This test confirmed skewness in creatinine levels in both males and females and within each age category for both males and females. Consequently, the study employed a non-parametric technique by determining the median and the 95th percentile intervals. A *p*-value below 0.05 was deemed statistically significant.

Creatinine concentration reference intervals were divided into 18 age groups, namely 0–<1 year, 1–<2 years, 2–<3 years, 3–<4 years, 4–<5 years, 5–<6 years, 6–<7 years, 7–<8 years, 8–<9 years, 9–<10 years, 10–<11 years, 11–<12 years, 12–<13 years, 13–<14 years, 14–<15 years, 15–<16 years, 16–<17 years, and 17–<18 years.

**Figure 1:**
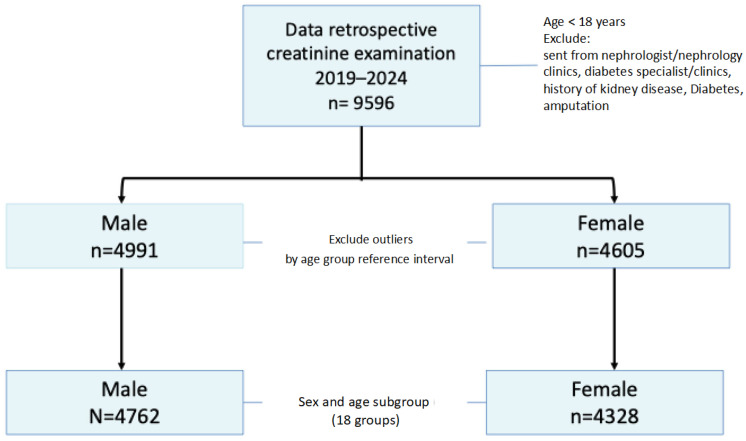
Flowchart study enrollment.

From a dataset of 4991 males and 4605 females, we eliminated outliers in each age group utilizing Box–Cox transformation and Tukey’s method (1977). These outliers were excluded from further computations or estimations of the reference interval for each age group, resulting in 4762 (52.38%) males and 4328 (47.65%) females (Figure 1). The outlier detection approach entails calculating the lower and upper quartiles, which correspond to the 25th (Q1) and 75th (Q3) percentiles, respectively, of the dataset. The lower boundary is equal to Q1 minus 1.5 times the interquartile range (IQR), and the upper boundary is equal to Q3 plus 1.5 times the IQR. An outlier refers to any data point that falls outside the established boundaries, either below the lower boundary or above the upper boundary [13].

The data were presented as the median and interquartile range (IQR) for continuous variables. Reference intervals were established based on transformed data when the data did not follow a normal distribution. The study established the median and percentiles P2.5 (lower reference limit/LRL) and P97.5 (upper reference limit/URL). Each group’s P97.5 values were the serum creatinine URLs based on age and gender [8]. Due to limited data in certain age groups, median creatinine comparison analysis between regions was calculated by categorizing ages according to the Eunice Kennedy Shriver National Institute of Child Health and Human Development (NICHD). The age groups included were <1 year, 1–<4 years, 4–<12 years, 12–<16 years, and 16–<18 years. The Mann–Whitney U test was used to assess differences between age groups and also across regional categories throughout each age range, with significance defined as *p* < 0.05. Statistical analyses were conducted using MedCalc^®^ software for Windows (version 20.218).

## 3. Results

**Figure 2:**
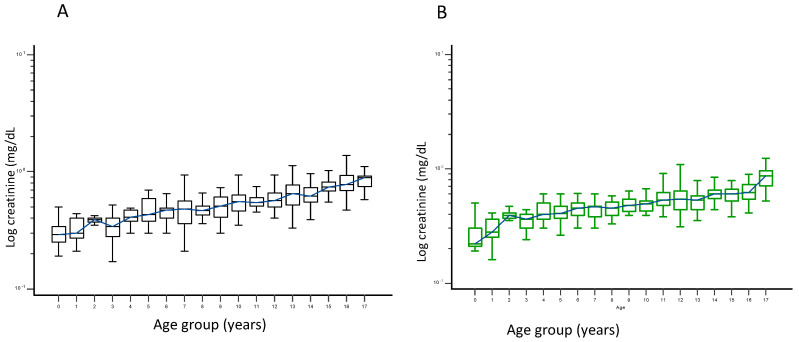
Male (**A**) and female (**B**) creatinine concentration reference intervals in different age groups. Note: 0 = 0–<1 year, 1 = 1–<2 years, 2 = 2–<3 years, 3 = 3–<4 years, 4 = 4–<5 years, 5 = 5–<6 years, 6 = 6–<7 years, 7 = 7–<8 years, 8 = 8–<9 years, 9 = 9–<10 years, 10 = 10–<11 years, 11 = 11–<12 years, 12 = 12–<13 years, 13 = 13–<14 years, 14 = 14–<15 years, 15 = 15–<16 years, 16 = 16–<17 years, and 17 = 17–<18 years.

We established serum creatinine URLs for children according to their ages and sexes (Table 1 and Table 2). Both males and females experience a gradual rise in creatinine levels as they age (Figure 2A,B). During adolescence, a notable increase was observed, typically around ages 13–14 and 15–18 for males, whereas females experience a gradual increase that becomes pronounced after ages 16–18 (Figure 2A,B). In males, the age cohorts of 5–<6 years, 6–<7 years, and 7–<8 years exhibited no variation in median blood creatinine levels (*p* > 0.05); a similar observation was seen in the cohorts of 10–<11 years and 11–<12 years, as well as in the age groups of 13–<14 and 14–<15 years (*p* > 0.05) (Table 1). In females, median blood creatinine levels exhibited no variation between the 4–5 and 5–6 age groups, the 6–<7, 7–<8, and 8–<9 age groups, the 9–<10 and 10–<11 age groups, as well as among the 11–<12, 12–<13, and 13–<14 age groups (*p* > 0.05) (Table 2).

In addition, the age stratification did not consistently show increasing creatinine serum URLs with age. For example, in males aged 1–2, 2–3, 3–4, 4–5, 5–6, and 6–7 years, the URLs were 0.60, 0.45, 0.58, 0.61, 0.91, and 0.63 mg/dL, respectively (Table 1).

In males aged 1–<4 years, median blood creatinine levels exhibited no regional variation (*p* > 0.05), but significant differences were seen across the three areas in other age groups (*p* < 0.05) (Table 3). In females across all age groups, substantial disparities in median creatinine levels were seen among the three areas, except in the 4–<12 age group, where no variations in median serum creatinine were noted between the Java and Sulawesi regions, as well as between the Sulawesi and Sumatra regions. A similar occurrence transpired in the Java and Sulawesi regions in the age ranges of 12–<16 years and 16–<18 years (Table 4).

## 4. Discussion

Serum creatinine is a frequently tested substance in clinical chemistry laboratories around the world [12]. Although it has limitations, this endogenous marker examination is less costly compared to other substances used to assess kidney function [14]. The measurement of creatinine serum concentration is highly favourable in assessing kidney damage, establishing appropriate treatment dosages, and measuring the GFR to diagnose CKD [9,10,11]. Reference values for serum creatinine, categorised by age and sex, enable clinical laboratories to determine normal renal function in paediatric patients without the need for height information [15]. Pediatric creatinine reference data for individuals up to 18 years of age has been conspicuously absent, creating a significant clinical and laboratory gap. This study addresses this critical need, providing invaluable data for both laboratories and clinicians interpreting pediatric creatinine values. These newly established reference intervals serve as a crucial first-line indicator for potential renal dysfunction. Critically, even creatinine elevations remaining within population-derived “normal” ranges may herald incipient renal compromise, underscoring the importance of these reference values for early detection and intervention [12].

Jhee and colleagues [16] found that URL blood creatinine concentrations may predict chronic kidney disease, based on fourteen years of Korean cohort data. Their findings underscore the critical importance of careful interpretation of serum creatinine levels, even within the conventionally defined normal range. This is due to the upper limit of normal serum creatinine being associated with an increased risk of CKD progression. This phenomenon, known as the creatinine-blind range, can be attributed to the compensatory mechanism of augmented tubular secretion of creatinine in individuals with mildly compromised renal filtration. Consequently, early impairment of tubular creatinine secretion can lead to elevated serum creatinine levels, serving as a harbinger of CKD [16,17].

Significant differences in creatinine levels exist across age groups and regions for both males and females. Age, gender, and ethnicity are among the variables that may influence creatinine level variations [12]. This means that the same blood creatinine levels may indicate different GFR levels. Given the importance of creatinine levels in evaluating renal function, it is imperative to use a cautious approach when interpreting blood creatinine levels, especially in children, due to the impact of factors like age and variations in muscle mass.

In laboratory practices, the reference intervals used may be derived from textbooks, research journals, or national references, and if there are none, the laboratory usually uses manufacturer data. In blood creatinine examination, the reference value for children that is widely used by paediatricians, from textbooks such as *Nelson*, which combine age ranges, is certainly not appropriate to use. For example, for the age group 0–4 years, the creatinine reference in males is 0.5 mg/dL [18]. In this study, there was a difference in median creatinine between those ages (range from 0.22 to 0.4 mg/dL) that also differed from other studies (<0.48 mg/dL) [19,20].

As observed in this study, there were differences in creatinine levels with each increase in the age range of the cohort group, while some did not increase with age range. In several age groups, creatinine URLs were found to fluctuate, mainly at ages 1–7. Similar findings were obtained in studies by Savory (age 1–8) [21] and Ahmed (age 3–5) [22]. The study showed that serum creatinine levels in males rise noticeably around ages 13–14 (0.65 mg/dL) and ages 15–18 (0.89 mg/dL). In females, the rise was slower at first but became noticeable after ages 16–18 (0.87 mg/dL). This observation is in line with findings by GT Chuang from a single-centre hospital population in Taiwan, which had a range measured in mg/dL that increased from age 12 (0.68 mg/dL) to age 17 (0.84 mg/dL) [8].

No variations in blood creatinine levels were observed throughout several age cohorts, including males aged 5–8 years and those aged 10–15 years. It is suggested that these specific 1-year intervals be combined into slightly broader, more stable groups for clinical application without losing precision.

A considerable rise in creatinine levels was noted in the 15–18 age group. Similar phenomena occur in females, where substantial disparities at the age of 15 may be associated with dietary status and growth spurts [18,23,24]. However, in this study we were limited to evaluating nutritional status and muscle mass.

Age and gender variations in creatinine production have led to extensive research on establishing population-specific normal reference ranges. Pottel and colleagues conducted detailed studies to determine age- and gender-specific intervals from neonates to elderly Caucasians [20]. However, there was a lack of data on other ethnicities as well as Asian populations, especially in children. This may result from variations in demographic characteristics between countries, and the interpretation of creatinine levels is more complex than anticipated [12]. This study revealed a considerable disparity in blood creatinine levels among three geographical zones. Varying dietary habits (e.g., protein intake), average muscle mass across regions, or even subtle differences in environmental factors might influence population health and, consequently, creatinine levels. Therefore, establishing region-specific reference intervals for creatinine is likely to be beneficial.

There was a significant rise in levels of serum creatinine observed in individuals aged 15 to 17, with a more pronounced effect in males. This study aligns with the findings of Suwanrungroj et al. and Chuang et al., who observed an elevation in serum creatinine levels with age, particularly in individuals aged 15–16 [8,15]. The findings revealed that blood creatinine concentrations in the reference period consistently rise with age, with males experiencing greater elevation than females during adolescence. This phenomenon can be attributed to the more rapid increase in muscle mass in men compared to women [25]. Studies have demonstrated a disparity in muscle mass between males and females among children, adolescents, and adults, with a notable association with creatinine levels [24,26]. The use of reference interval data generated before the standardization of creatinine calibrators (2006, Isotop dilution mass spectrometry/IDMS) and different creatinine testing methods (Jaffe versus enzymatic) needs careful thought and attention. In this study, we employed a traceability calibrator for IDMS. The Abbott enzymatic examination manufacture insert kit used in this study only presented the normal range for the adult population, not for children. Therefore, this reference interval research is highly beneficial for physicians and laboratories.

While laboratory validation of reference intervals, as described by Ozardo [27], is crucial, this study encountered challenges in procuring data specifically from healthy children, a common hurdle in pediatric research. The observed significant inter-age group variability underscores the need for larger sample sizes to refine these intervals further. Although the indirect methodologies employed here for reference interval establishment may present inherent limitations, they offer a pragmatic and statistically sound approach when working with large, heterogeneous datasets like the one analysed in this study [28]. Future research with expanded cohorts will further enhance the precision and applicability of these vital pediatric reference intervals.

This study holds significant value due to its assessment of creatinine levels across three of Indonesia’s most populous regions. The research demonstrates that, even when employing standardized instrumentation and methodologies, creatinine levels exhibit regional variation.

Qcr in adults has been extensively researched [29]; however, its occurrence in children, particularly across multi-region areas like Indonesia, is still unknown. In populations with a wide range of ethnicities, such as Indonesia (300 ethnicities and inter-ethnic marriages), it is imperative to establish a Qcr that can be used to apply the eGFR formula regardless of the individual’s race. This is important, as the topic of race is a delicate matter and is taken into account. For instance, Delayane et al. revealed distinct traits between European and American Black races, causing the definition of Black in American and European contexts to be questionable [12,30,31].

## 5. Limitations

The lack of an integrated health database in Indonesia hinders the availability of comprehensive national health data. This is understandable, because obtaining a creatinine examination in the children population’s is difficult. In addition to the financial constraints preventing payment for laboratory examinations (which are not covered by government health insurance), there are also challenges arising from the fact that many children who are technically healthy decline routine laboratory screening examinations. Certain data within the age group failed to meet the minimum requirement of 120 entries per age–region category, necessitating additional study to obtain more thorough information. Since the study only includes populations from three Indonesian islands, this does not represent true ethnic diversity or a “multi-population” cohort. The generalizability of the findings in this study is limited. This work has limitations owing to the absence of external validation. Consequently, prospective research must be conducted to ensure the generalizability of the study results. Also, the decision to use broader age groupings for regional comparison due to limited data on certain age groups is understandable, but it may lead to potential heterogeneity within those broader groups.

Moreover, the authors were unable to minimize the potential influence of factors affecting creatinine levels due to the limited exclusion of data—for example, the inclusion of children who are professional bodybuilders, athletes, or have eating problems that may affect muscle mass and subsequently affect creatinine values, as well as the potential influence of nonsteroidal anti-inflammatory medicines (NSAIDs) or H2 blockers on creatinine levels. Also, characteristics such as height and weight can potentially be evaluated in the future.

## 6. Conclusions

Stratification of clinical laboratory data by age and sex enabled the establishment of pediatric reference intervals and corresponding Qcr levels. Analysis revealed age-dependent variations in reference ranges, demonstrating a progressive increase in both upper and lower limits up to 18 years of age. These newly derived, age- and sex-specific reference values provide a crucial resource for clinical laboratories, empowering clinicians to more accurately assess pediatric renal function. Consequently, this refined diagnostic approach facilitates timely and precise diagnoses, ultimately leading to improved clinical outcomes for children with renal disorders. Further investigation is warranted for the comparison of mGFR to eGFR when using a non-race-based formula based on median (Q) creatinine in children population. Given the substantial regional disparities, there is a need for region-specific intervals. Therefore, further research is essential to establish the reference interval and median for each population.

## Figures and Tables

**Table 1:** Reference intervals of serum creatinine in males.

Age (Year)	n	Median (mg/dL)	IQR	Percentile (mg/dL)				*p* Value
				25th	75th	2.5th (LRL)	97.5th (URL)	
<1	121	0.29	0.09	0.25	0.34	0.19	0.50	
1–<2	216	0.30	0.13	0.27	0.40	0.21	0.83	0.0065
2–<3	168	0.39	0.03	0.37	0.40	0.35	0.45	<0.0001
3–<4	391	0.34	0.12	0.28	0.40	0.17	0.51	<0.0001
4–<5	310	0.41	0.09	0.38	0.47	0.30	0.48	<0.0001
5–<6	345	0.43	0.21	0.38	0.59	0.30	0.70	<0.0001
6–<7	257	0.47	0.09	0.40	0.49	0.30	0.65	0.3910
7–<8	172	0.48	0.20	0.36	0.56	0.23	0.71	0.7309
8–<9	240	0.47	0.09	0.43	0.51	0.36	0.77	0.0265
9–<10	180	0.51	0.20	0.41	0.61	0.30	0.73	0.0054
10–<11	204	0.56	0.17	0.46	0.63	0.35	0.94	0.0161
11–<12	152	0.55	0.09	0.51	0.60	0.46	0.75	0.2251
12–<13	276	0.57	0.16	0.50	0.66	0.40	0.91	0.1606
13–<14	255	0.65	0.25	0.52	0.77	0.39	0.93	<0.0001
14–<15	246	0.62	0.19	0.55	0.73	0.40	0.94	0.2320
15–<16	160	0.74	0.13	0.69	0.82	0.55	1.02	<0.0001
16–<17	375	0.78	0.24	0.69	0.93	0.47	1.20	0.0337
17–<18	694	0.89	0.17	0.75	0.92	0.60	1.04	<0.0001
Total	4762							

Note: The Mann–Whitney U test was used to assess differences between age groups. Abbreviations: LRL, lower reference limit; URL, upper reference limit. The calculated *p*-value represents the statistical significance between sequential age groups (age < 1 and 1–2, age 1–2 and age 2–3, etc.).

**Table 2:** Reference intervals of serum creatinine in females.

Age (Year)	n	Median (mg/dL)	IQR (mg/dL)	Percentile (mg/dL)				*p* Value
				25th	75th	2.5th (LRL)	97.5th (URL)	
<1	199	0.22	0.11	0.19	0.30	0.19	0.50	
1–<2	208	0.28	0.11	0.25	0.36	0.16	0.41	<0.0001
2–<3	209	0.39	0.06	0.35	0.41	0.35	0.47	<0.0001
3–<4	297	0.36	0.10	0.30	0.40	0.19	0.44	<0.0001
4–<5	245	0.40	0.14	0.36	0.50	0.30	0.60	<0.0001
5–<6	358	0.40	0.10	0.37	0.47	0.26	0.60	0.4613
6–<7	243	0.45	0.11	0.39	0.50	0.26	0.61	<0.0001
7–<8	240	0.47	0.09	0.38	0.47	0.30	0.60	0.2610
8–<9	177	0.45	0.12	0.39	0.51	0.33	0.58	0.2986
9–<10	195	0.48	0.12	0.42	0.54	0.39	0.64	0.0001
10–<11	192	0.49	0.10	0.43	0.52	0.39	0.67	0.9129
11–<12	205	0.53	0.14	0.48	0.62	0.38	0.84	<0.0001
12–<13	217	0.54	0.20	0.44	0.64	0.31	1.16	0.6283
13–<14	210	0.53	0.14	0.44	0.58	0.35	0.79	0.2225
14–<15	192	0.60	0.11	0.55	0.65	0.44	0.84	<0.0001
15–<16	194	0.60	0.14	0.52	0.66	0.33	0.79	0.9497
16–<17	315	0.64	0.19	0.54	0.73	0.41	1.29	0.0076
17–<18	432	0.87	0.25	0.71	0.96	0.56	1.11	<0.0001
Total	4328							

Note: The Mann–Whitney U test was used to assess differences between age groups Abbreviations: LRL, lower reference limit; URL, upper reference limit. The calculated *p*-value represents the statistical significance between sequential age groups (age < 1 and 1–2, age 1–2 and age 2–3, etc.).

**Table 3:** Comparison of male Q creatinine values among three regions.

Age	Region	n	Median (mg/dL)	IQR (mg/dL)	*p* Value *
**<1 year**	Java	68	0.4	0.25–0.35	<0.0001 ^a^
	Sulawesi	56	0.58	0.17–0.30	
	N/A				
**1–<4 year**	Java	1281	0.44	0.34–0.83	0.4437 ^a^
	Sulawesi	97	0.58	0.40–0.63	0.9226 ^b^
	Sumatera	519	0.47	0.38–0.80	0.1506 ^c^
**4–<12 year**	Java	1946	0.55	0.44–0.72	0.0170 ^a^
	Sulawesi	189	0.54	0.47–0.61	<0.0001 ^b^
	Sumatera	433	0.66	0.60–0.89	<0.0001 ^c^
**12–<16 year**	Java	858	0.72	0.59–0.80	<0.0001 ^a^
	Sulawesi	90	0.60	0.56–0.69	<0.0001 ^b^
	Sumatera	93	0.74	0.64–0.97	0.0013 ^c^
**16–<18 year**	Java	446	0.89	0.79–0.89	<0.0001 ^a^
	Sulawesi	38	0.63	0.58–0.64	<0.0001 ^b^
	Sumatera	234	0.86	0.70–0.93	0.0004 ^c^

* Note: The Mann–Whitney test was used to compare the medians: Java vs. Sulawesi = ^a^, Sulawesi vs. Sumatera = ^b^, and Java vs. Sumatera = ^c^. N/A = not available. The results are considered significant if the *p*-value is less than 0.05.

**Table 4:** Comparison of female Q creatinine values among three regions.

Age	Region	n	Median (mg/dL)	IQR (mg/dL)	*p* Value *
**<1 year**	Java	93	0.22	0.21–0.23	
	Sulawesi	N/A			
	Sumatera	39	0.30	0.24–0.37	<0.0001 ^c^
**1–<4 year**	Java	741	0.37	0.31–0.41	<0.0001 ^a^
	Sulawesi	45	0.53	0.53–0.59	<0.0001 ^b^
	Sumatera	243	0.35	0.28–0.40	0.0010 ^c^
**4–<12 year**	Java	1393	0.47	0.41–0.53	<0.0001 ^a^
	Sulawesi	212	0.43	0.42–0.53	0.0602 ^b^
	Sumatera	222	0.46	0.39–0.53	0.1179 ^c^
**12–<16**	Java	817	0.60	0.53–0.69	0.0181 ^a^
	Sulawesi	90	0.55	0.50–0.67	0.2635 ^b^
	Sumatera	95	0.51	0.64–0.97	0.0005 ^c^
**16–<18**	Java	357	0.70	0.57–0.88	0.0004 ^a^
	Sulawesi	34	0.81	0.70–0.95	0.29791 ^b^
	Sumatera	203	0.78	0.65–0.92	<0.0001 ^c^

* Note: The Mann-Whitney test was used to compare the medians: Java vs. Sulawesi = ^a^, Sulawesi vs. Sumatera = ^b^, and Java vs. Sumatera = ^c^. N/A = not available. The results are considered significant if the *p*-value is less than 0.05.

## Data Availability

The datasets presented in this article are not readily available because the data are part of an ongoing study. Requests to access the datasets should be directed to aryati@gmail.com.
